# Association between dietary insulin index and load with brain derived neurotrophic factor, adropin and metabolic health status in Iranian adults

**DOI:** 10.1038/s41598-023-48056-x

**Published:** 2023-11-23

**Authors:** Roxana Nematbakhsh, Zahra Hajhashemy, Keyhan Lotfi, Farnaz Shahdadian, Parisa Rouhani, Parvane Saneei

**Affiliations:** 1 Department of Community Nutrition, School of Nutrition and Food Science, Nutrition and Food Security Research Center, Isfahan University of Medical Sciences, PO Box 81745-151, Isfahan, Iran; 2 Students’ Research Committee, Isfahan University of Medical Sciences, Isfahan, Iran; 3 Department of Community Nutrition, School of Nutritional Sciences and Dietetics, Tehran University of Medical Sciences, Tehran, Iran; 4 Department of Clinical Nutrition, School of Nutrition and Food Science, Food Security Research Center, Isfahan University of Medical Sciences, Isfahan, Iran

**Keywords:** Nutrition, Lifestyle modification

## Abstract

The associations of high potential insulinogenic foods with metabolic health (MH) status and brain-derived neurotrophic factor (BDNF) and adropin were not investigated quite enough. We examined the relationship between dietary insulin load (DIL) and dietary insulin index (DII) with MH and serum levels of BDNF and adropin among Iranian adults. This cross-sectional investigation accomplished among 527 Iranian middle-aged adults (54.3% men). Dietary information was obtained by a validated food frequency questionnaire. Anthropometric indices and blood pressure were assessed. For measuring lipid and glycemic profile and serum levels of BDNF and adropin, blood samples were assembled after 12 h of fasting. MH was defined based on lipid and glycemic profile, high blood pressure, insulin resistance and chronic inflammation. After adjustments all confounders, participants in the highest tertile of DII compared to the lowest one had a 115% increased odds for metabolic unhealthy (MU) profile (OR_T3 vs. T1_ = 2.15, 95% CI 1.03–4.49). However, DIL was not related to MU. Higher DII was additionally associated with high blood pressure, in maximally-adjusted model (OR_T3 vs. T1_ = 3.57, 95% CI 1.61–7.92). Moreover, moderate DIL was significantly associated with hypertriglyceridemia (OR_T2 vs. T1_ = 2.56, 95% CI 1.01–6.45). Each tertile increase in DII or DIL was not significantly associated with serum BDNF or adropin values. Greater DII was associated with higher chance of MU and hypertension in Iranian adults; but no association was found between DIL and metabolic health. DIL or DII was not related to circulating BDNF or adropin. To confirm these findings, additional prospective investigations are required.

## Introduction

Obesity and overweight have become so prevalent among adults worldwide, and are two of the most important concerns these days^1^. Several cardiovascular risk factors such as hypertension, insulin resistance and hypertriglyceridemia are usually associated with obesity and overweight^2^. However, all individuals with obesity do not have these metabolic abnormalities. Individuals with obesity can be metabolically healthy or unhealthy^3,4^. The worldwide prevalence of metabolically healthy (MH) in adults with obesity and metabolically unhealthy (MU) among those with normal weight is 7.27% and 19.98%, respectively^5^. The prevalence of metabolically healthy obese (MHO) in Iranian subjects is 7.5%^6^ and the prevalence of metabolically unhealthy normal weight (MUNW) among Iranian individuals is 17.2%^7^. The risk of progressing metabolic abnormalities-related diseases is lower in MH individuals compared to MU subjects^8^.

In spite of the role of genetic factors and heredity, some other factors (such as adipose tissue function, chronic stress, cardio-respiratory fitness and lifestyle) were proposed as determinants of MH status^9^. Moreover, it seems that chronic inflammation is associated with MH status^10^. Some previous studies have demonstrated that insulin resistance is leading to chronic inflammation^11^. A diet, that leads to an increase in blood sugar, is seem to be the major risk factor for insulin resistance, because it would potentially affect post-prandial insulin and lead to insulin resistance^12,13^. Dietary insulin index (DII) is referring to postprandial insulin secretion after eating common foods compared to a isoenergetic reference food^14^ Dietary insulin load (DIL) is another definition that is calculating through DII of each food and its energy^15^.

Brain-derived neurotrophic factor (BDNF), a member of the neurotrophic growth family, has recently considered as a mediator for reducing the risk of cardiovascular disease (CVD), type 2 diabetes mellitus (T2DM), hyperglycemia, dyslipidemia, obesity, and metabolic syndrome (MetS)^16^. In addition, adropin is a short peptide hormone, which is expressed in various organs and tissues such as liver, heart, and many parts of the brain, is associated with metabolic disorders. This protein participates in several biological activities (including metabolism of macronutrients and energy homeostasis) and can be affected by dietary components^17^.

Some previous studies have shown that DII and DIL were associated with MetS and other metabolic disorders such as obesity and T2DM^18,19^. One prospective study in Iran has shown a marginal positive association between DII and insulin resistance; elevated DIL was additionally linked with increased risk of insulin resistance after 3-year of follow-up^15^. Another prospective investigation among Iranian population has revealed that DII and DIL were associated with weight gain, but not with MetS^20^. A cross-sectional study in Ravansar in western Iran has indicated that higher DII and DIL were associated with both increased risk of MetS and abdominal obesity in patients with type 2 diabetes^21^. Another cross-sectional study among Iranian adolescent has shown that higher DII and DIL were significantly associated to greater chance of metabolically unhealthy obese (MUO), particularly in those with obesity^22^. However, no significant association was found between DII and DIL with MetS or obesity in another investigation on Iranian adults^23^ A cross-sectional analysis of the Shahedieh cohort study in Iran has also found a considerable positive relation between DII and MetS in women, but not in men^18^. Since no population-based investigation has studied the association of DII and DIL with serum BDNF, adropin and considering the contradictory findings of the previous studies with regard to MH, we conducted this study to examine the relation between DII and DIL with serum BDNF, adropin, and metabolic health status in Iranian adults.

## Materials and methods

### Study designs and participants

This cross-sectional study was conducted on a somewhat representative sample of adults in Isfahan, a main central city of Iran, in 2022. Totally, 600 qualified individuals were invited to participate in the study. A multistage cluster random-sampling method was used to choose 600 adults (from both genders) aged 20–60 years. For choosing a somewhat representative sample of the general adult population with different socioeconomic statuses, we have selected all adults who were working in 20 schools, including principals, teachers, school managers, assistants, other staffs and crews. However, individuals with the following features were not eligible: (1) being pregnant or lactating; (2) adhering to a specific diet; (3) having a previous history of stroke, cardiovascular disease, type 1 diabetes, and cancer. Among invited individuals, 543 of them accepted to take part in our study (response rate: 90.5%). We excluded subjects with the subsequent criteria: (1) had left more than 40 items of the food frequency questionnaire blank (n = 4); (2) reported a total energy intake out of the range of 800–4200 kcal/day (as under-reporters and over-reporters of total energy intake) (n = 3); (3) did not have data of their blood pressure (BP) measurement (n = 8); and (4) did not accept blood draw (n = 1). Finally, the analyses were run on data of 527 adults. Written informed agreements were taken from all participants. The procedure of the study was ethically approved by the local Ethics Committee of Isfahan University of Medical Sciences (no. 2402110). All methods were performed in accordance with the relevant guidelines and regulations (STROBE).

### Assessment of dietary intakes

We assessed the long-term dietary intake of participants by a validated Willett-format semi-quantitative 168-item food frequency questionnaire (FFQ)^24^. A former validation research of this FFQ on 132 middle-aged adults demonstrated sensible correlations between dietary intakes assessed through FFQ and those acquired from several 24-h dietary recalls (24 h-DR)^24^. The correlation coefficients between the dietary intakes from the FFQ and those from the twelve 24 h-DRs were 0.55 for total energy, 0.65 for proteins, 0.59 for fat, 0.67 for fiber, and 0.65 for magnesium. The reliability of the FFQ was assessed through comparing nutrient intakes obtained from this FFQ in two different occasions, 1 year apart. Overall, these data demonstrate that this FFQ could be valid and reliable for measuring common dietary intakes of Iranian adults^24^. An expert dietitian instructed the participants of the study to complete the FFQ through reporting the amount and frequency of their food intakes in the past year. Then, the portion sizes of consumed foods were changed to g/day by using common household measurements. Afterwards, all food items were entered into Nutritionist IV software, to acquire total daily energy and nutrients intake.

### Assessment of dietary insulin index and dietary insulin load

We applied food insulin index (FII) that shows the area under the curve of increased insulin during 2 h after eating a 1000 kJ portion of the test food divided by the area under the curve after consumption of a 1000 kJ of the reference food. The FII for every single food was provided through the former investigations of Holt et al.^14^, Bao et al.^25^, Bell et al.^26^ and Sadeghi et al.^18^ which supplied a wide list of FII. For any food item that its FII was not reported in these publications, FII of similar food was used. The insulin load for each food item was calculated through following formula:

Insulin load for a food item = insulin index of that food × amount of energy per 1 g of that food (g/d) × quantity of that food consumed (g/d)^27^. DIL for each individual was calculated by summing the insulin load of all consumed food items. Afterward, DII for each person was calculated through dividing DIL by total energy intake.

### Assessment of anthropometric, blood pressure and biochemical indices

Two expert dietitians measured height by using a stadiometer, while participants were standing with minimum clothing and without shoes, with an accuracy close to 0.1 cm. Body weight was measured through a body composition analyzer (TanitaMC-780MA, Tokyo, Japan). Body mass index (BMI) was calculating by Quetelet formula through dividing weight (kg) by the square of height (m^2^). For measuring waist circumstances (WC), a non-stretchable tape was used. After a normal aspiration, WC was measured at the narrowest area between lower rib and iliac crest without applying any pressure to the surface of the body. Then, WC measurement of each participant was repeated and the average of two measurements was applied in the analysis. Also, systolic and diastolic blood pressure (BP) was measured twice, by using a digital sphygmomanometer (OMRON, M3, HEM-7154-E, Japan) on the left arm, with a 0.5 mmHg precision. Before BP measurements, each participant was sitting for five minutes, did not drink tea or coffee and his/her bladder was empty. The mean of two measurements was recorded for each individual. For assessing biochemical indices, we collected blood samples after 12 h of fasting and let them to get coagulated. Then, samples were centrifuged to separate serum. Fasting blood glucose (FBG), TG and HDL-c were measured by using Biosystem A15 auto-analyzer and by glucose oxidase, glycerol phosphate oxidase and cholesterol oxidase methods, respectively. In order to evaluate serum levels of high sensitivity C-reactive protein (hs-CRP) and insulin, commercial kits (turbidimetry kit and latex enhanced turbidimetric method, Delta.DP; and Monobined Inc. Lake Forest, CA 92630, USA, respectively) were used. To determine the Homeostasis Model Insulin Resistance (HOMA-IR), the following formula was used^28^: HOMA-IR = [Fasting glucose (mg/dL) × fasting insulin(μU/mL)]/405.

For measuring serum BDNF and adropin amounts, ELISA kits (Zellbio, Veltlinerweg, Germany) were used.

### Assessment of metabolic health status

According to the method proposed by Wildman et al.^29^, the participants were divided into categories of metabolically healthy (MH) and metabolically unhealthy (MU). Based on this method, the participants with two or more of the following risk factors, were considered as MU: (1) fasting glucose level ≥ 100 mg/dL or using antidiabetic drugs; (2) HDL-c level < 40 mg/dL in men or < 50 mg/dL in women; (3) serum triglyceride level ≥ 150 mg/dL; (4) systolic/diastolic blood pressure ≥ 130/85 mmHg or using antihypertensive drugs; (5) HOMA-IR > 90th percentile; and (6) hs-CRP level > 90th percentile. Those with only one or none of the above-mentioned criteria were considered as MH^29^.

### Assessment of other variables

We used the validated International Physical Activity Questionnaires (IPAQ) questionnaire to assess the individual’s physical activity levels^30^. Also, we collected data of other confounding variables such as age, sex, education, marital status, socio-economical status (SES), smoking habits, medical background of diseases and drug use through a pre-tested self-reported questionnaire.

### Statistical analysis

A minimum sample of 474 individuals were required for the current study by considering type 1 error of 0.05, power of 80%, precision (d) of 4.5% and a prevalence of 49.4% for having a metabolic unhealthy profile^31^. Based on Kolmogorov–Smirnov test, all quantitative variables had normal distribution. Mean ± SD/SE and number (percentage) for continuous and categorical variables were respectively reported. First, participants divided into tertiles of DIL and DII. Then, for comparing the distribution of categorical and continuous variables across teriles of DIL and DII, chi-square test and one-way analysis of variance (ANOVA) were applied. For reporting sex, age and energy-adjusted dietary intakes of individuals across tertiles of DIL and DII, analysis of covariance (ANCOVA) was used. To compute odd ratios (OR) of MU across tertiles of DIL and DII, binary logistic regression was applied in crude and multivariable-adjusted models. Age, gender and energy intake were adjusted in the first model. In the second model, education, marital status, smoking, socioeconomic status, physical activity levels, and dietary habits (including regular meal pattern, eating rate, intra meal fluid intake, salt and spices intake) were additionally controlled. Further adjustment for BMI was done in the third model. The reference category in all models was the first tertile of DIL and DII. In logistic regression models, tertiles of DIL and DII were considered as ordinal variables in order to obtain the trends. Linear regression was applied to compute the regression coefficient (B) for BDNF and adropin per one tertile increase in tertiles of DIL and DII, in crude and multivariable-adjusted models. For BDNF, the effects of sex and age were adjusted in the first model and the history of diabetes, depression, hypertension, and physical activity level were additionally controlled in the second model. For adropin, energy intake, sex and age were adjusted in the first model and the effect of smoking, BMI and physical activity level were additionally controlled in the second model. For all statistical analyses, SPSS software version 20 (SPSS, Inc., Chicago, IL, USA) was applied. *P*-values lower than 0.05 were assumed statistically significant.

### Ethical approval and consent to participate

All participants provided an informed written consent. The study protocol was approved by the local Ethics Committee of Isfahan University of Medical Sciences.

## Results

Participants of this study were 527 adults with a mean age of 42.66 (± 11.19) years; 54.3% of them (n = 286) were men. Among individuals, 42.5% (n = 224) were metabolically unhealthy. Demographic and cardiometabolic features of participants across tertiles of DIL and DII are presented in Table 1. Participants in the highest tertile of DIL, compared to the lowest one, had higher weight and WC and more likely to be male and had less fluid consumption with their meals. Individuals in the highest tertile of DII, compared to the lowest category, were more likely to be male and had lower socioeconomic status. No other significant difference was found between tertiles of DIL and DII.

**Table 1:** Demographic and cardiometabolic features of participants across tertiles of DIL and DII (n = 527)^1^.

Variables	Tertiles of DIL				Tertiles of DII^2^			
	Tertile 1 (n = 169) (< 79,808.09)	Tertile 2 (n = 179) (79,871.39–107,574.0)	Tertile 3 (n = 179) (> 107,574.0)	*P*-value	Tertile 1 (n = 170) (< 40.62)	Tertile2 (n = 177) (40.64–44.22)	Tertile3 (n = 180) (> 44.22)	*P*-value ^2^
Age (year)	43.09 ± 10.84	43.33 ± 11.21	41.58 ± 11.47	0.28	44.06 ± 10.86	42.25 ± 10.38	41.73 ± 12.15	0.13
Body weight (kg)	73.69 ± 12.92	74.04 ± 15.92	79.47 ± 14.04	< 0.001	74.30 ± 14.59	75.78 ± 15.19	77.15 ± 13.90	0.19
BMI (kg/m^2^)	26.85 ± 4.02	26.41 ± 5.05	27.47 ± 4.1	0.08	27.04 ± 4.74	26.85 ± 4.40	26.85 ± 4.19	0.90
WC (cm)	91.14 ± 10.46	91.64 ± 12.14	95.12 ± 11.41	0.01	91.70 ± 11.56	92.57 ± 11.60	93.67 ± 11.30	0.27
Sex				< 0.001				< 0.001
Male	76 (45.0)	91 (50.8)	119 (66.5)		69 (40.6)	89 (50.3)	128 (71.1)	
Female	93 (55.0)	88 (49.2)	60 (33.5)		101 (59.4)	88 (49.7)	52 (28.9)	
Education				0.21				0.67
Diploma or lower	20 (11.9)	14 (7.9)	24 (13.6)		17 (10.2)	18 (10.2)	23 (12.8)	
Higher than diploma	148 (88.1)	164 (92.1)	153 (86.4)		150 (89.8)	158 (89.8)	157 (87.2)	
Marital status				0.97				0.34
Single	27 (16.3)	27 (15.2)	31 (17.4)		29 (17.3)	26 (14.8)	30 (16.9)	
Married	137 (82.5)	148 (83.1)	145 (81.5)		135 (80.4)	150 (85.2)	145 (81.5)	
Divorced or widow	2 (1.2)	3 (1.7)	2 (1.1)		4 (2.4)	0 (0)	3 (1.7)	
Smoking				0.28				0.14
Non-smoker	143 (94.7)	146 (90.7)	151 (95.6)		141 (93.4)	143 (92.3)	156 (95.1)	
Ex-smoker	5 (3.3)	8 (5.0)	2 (1.3)		8 (5.3)	4 (2.6)	3 (1.8)	
Smoker	3 (2)	7 (4.3)	5 (3.2)		2 (1.3)	8 (5.2)	5 (3)	
SES				0.96				0.01
Low	35 (34)	35 (31.5)	36 (29.8)		32 (29.4)	33 (30)	41 (35.3)	
Moderate	31 (30.1)	37 (33.3)	39 (32.2)		25 (22.9)	37 (33.6)	45 (38.8)	
High	37 (35.9)	39 (35.1)	46 (38)		52 (47.7)	40 (36.4)	30 (25.9)	
Physical activity				0.26				0.17
Inactive	93 (55.7)	108 (60.3)	96 (53.9)		86 (50.6)	104 (59.4)	107 (59.8)	
Minimally active	64 (38.3)	60 (33.5)	62 (34.8)		69 (40.6)	62 (35.4)	55 (30.7)	
HEPA active	10 (6)	11 (6.1)	20 (11.2)		15 (8.8)	9 (5.1)	17 (9.5)	
Meal pattern				0.17				0.94
Regular	82 (51.3)	107 (61.1)	100 (58.8)		91 (56.2)	97 (58.1)	101 (57.4)	
Irregular	78 (48.8)	68 (38.9)	70 (41.2)		71 (43.8)	70 (41.9)	75 (42.6)	
Eating rate				0.07				0.42
Fast	16 (10.1)	23 (13.4)	14 (8)		20 (12.3)	16 (9.5)	17 (9.8)	
Moderate	117 (74.1)	129 (75)	147 (84.5)		118 (72.8)	136 (81.0)	139 (79.9)	
Slow	25 (15.8)	20 (11.6)	13 (7.5)		24 (14.8)	16 (9.5)	18 (10.3)	
Intra meal				0.02				0.13
Moderate	112 (70.9)	112 (68.3)	96 (56.8)		110 (71.0)	98 (60.1)	112 (64.7)	
More	46 (29.1)	52 (31.7)	73 (43.2)		45 (29.0)	65 (39.9)	61 (35.3)	
Salt and spices				0.37				0.30
High	14 (8.5)	15 (8.7)	21 (12.1)		21 (12.7)	19 (11.0)	10 (5.8)	
Moderate	123 (74.5)	128 (74.4)	133 (76.9)		120 (72.3)	129 (74.6)	135 (78.9)	
Low	28 (17)	29 (16.9)	19 (11)		25 (15.1)	25 (14.5)	26 (15.2)	
High BP				0.33				0.14
No	90 (53.3)	101 (56.4)	87 (48.6)		98 (57.6)	95 (53.7)	85 (47.2)	
Yes	79 (46.7)	78 (43.6)	92 (51.4)		72 (42.4)	82 (46.3)	95 (52.8)	
High FBG (≥ 100 mg/dL)				0.69				0.85
No	137 (81.1)	146 (81.6)	140 (78.2)		134 (78.8)	143 (80.8)	146 (81.1)	
Yes	32 (18.9)	33 (18.4)	39 (21.8)		36 (21.2)	34 (19.2)	34 (18.9)	
High TG (≥ 150 mg/dL)				0.31				0.22
No	115 (68)	110 (61.5)	109 (60.9)		113 (66.5)	116 (65.5)	105 (58.3)	
Yes	54 (32)	69 (38.50	70 (39.1)		57 (33.5)	61 (34.5)	75 (41.7)	
High hs-CRP				0.10				0.11
No	146 (86.4)	167 (93.3)	162 (90.5)		147 (86.5)	165 (93.2)	163 (90.6)	
Yes	23 (13.6)	12 (6.7)	17 (9.5)		23 (13.5)	12 (6.8)	17 (9.4)	
High HOMA-IR				0.76				0.48
No	150 (88.8)	162 (90.5)	163 (91.1)		150 (88.2)	163 (92.1)	162 (90.0)	
Yes	19 (11.2)	17 (9.5)	16 (8.9)		20 (11.8)	14 (7.9)	18 (10.0)	
Low HDL-c (< 40/50 mg/dL)				0.72				0.38
No	152 (89.9)	158 (88.3)	156 (87.2)		154 (90.6)	152 (85.9)	160 (88.9)	
Yes	17 (10.1)	21 (11.7)	23 (12.8)		16 (9.4)	25 (14.1)	20 (11.1)	
Adropin (pg/mL)	59.86 ± 47.90	55.43 ± 31.52	54.74 ± 40.16	0.47	55.46 ± 36.64	55.67 ± 38.96	58.48 ± 44.22	0.74
BDNF (ng/mL)	1.19 ± 0.49	1.45 ± 2.73	1.11 ± 0.61	0.14	1.15 ± 0.51	1.49 ± 2.75	1.11 ± 0.54	0.06

^1^Quantitative variables mean ± SD. Qualitative variables: frequency (percentage).

^2^Resulted from ANOVA for quantitative variables and chi-square test for categorical variables.

Abbreviations: *DIL* Dietary insulin load, *DII* Dietary insulin index, *BMI* Body mass index, *WC* Waist circumstance, *SES* Socio-economical status, *HEPA* Health-enhancing physical activity, *BP* Blood pressure, *FBG* Fasting blood glucose, *TG* Triglyceride, *hs-CRP* high sensitivity C-reactive protein, *HOMA-IR* Homeostasis model insulin resistance, *HDL-c* High-density lipoprotein cholesterol, *BDNF* brain-derived neurotrophic factor.

Multivariable-adjusted dietary intakes (energy, macronutrients, and food items) of study participants across tertiles of DIL and DII are presented in Table 2. People in the top tertile of DIL, compared to the bottom tertile, had a higher intake of energy and grains, and lower intake of meat, nuts, mono-unsaturated fatty acids (MUFA), poly-unsaturated fatty acids (PUFA), saturated fatty acids (SFA), cholesterol, and protein. Participants in the highest category of DII, compared to the lowest category, had more consumption of grains and carbohydrates; in contrast, they had less vegetable, meat, nuts, dairy, MUFA, PUFA, SFA, cholesterol, protein, and fat intake.

**Table 2:** Multivariable-adjusted dietary intakes (energy, macronutrients and food items) of study participants across tertiles of DIL and DII (n = 527)^1^.

	Tertiles of DIL				Tertiles of DII			
	T_1_ (n = 169)	T_2_ (n = 179)	T_3_ (n = 179)	*P*^2^	T_1_ (n = 170)	T_2_ (n = 177)	T_3_ (n = 180)	*P*
Energy (Kcal/d)	1568.36 ± 27.03	2206.88 ± 26.14	3016.09 ± 26.45	< 0.001	2317.96 ± 52.88	2273.43 ± 50.97	2241.75 ± 51.70	0.60
Food groups (g/day)								
Fruits	603.98 ± 37.42	557.39 ± 23.94	509.34 ± 38.01	0.37	540.97 ± 24.83	550.20 ± 23.92	575.94 ± 24.27	0.59
Vegetables	392.65 ± 26.20	347.12 ± 16.76	287.56 ± 26.62	0.08	397.24 ± 17.14	339.18 ± 16.52	291.10 ± 16.76	< 0.001
Meats	117.25 ± 6.78	101.19 ± 4.33	79.07 ± 6.88	0.01	111.61 ± 4.43	103.94 ± 4.27	81.72 ± 4.33	< 0.001
Fish	9.74 ± 1.08	7.54 ± 0.69	5.53 ± 1.1	0.09	8.17 ± 0.72	7.71 ± 0.69	6.85 ± 0.70	0.43
Legumes	35.70 ± 4.31	37.89 ± 2.76	43.96 ± 4.38	0.51	37.69 ± 2.86	38.80 ± 2.75	41.17 ± 2.79	0.68
Nuts	16.48 ± 1.46	13.44 ± 0.93	5.85 ± 1.48	< 0.001	16.37 ± 0.95	11.52 ± 0.91	7.87 ± 0.93	< 0.001
Grains	285.76 ± 19.00	374.34 ± 12.15	492.73 ± 19.30	< 0.001	323.95 ± 12.52	389.91 ± 12.06	441.19 ± 12.24	< 0.001
Dairy	336.85 ± 31.22	305.06 ± 19.97	309.29 ± 31.71	0.65	368.34 ± 20.41	329.77 ± 19.66	255.04 ± 19.95	< 0.001
MUFA	27.13 ± 0.75	22.54 ± 0.48	15.91 ± 0.76	< 0.001	25.52 ± 0.47	22.01 ± 0.46	17.97 ± 0.46	< 0.001
PUFA	20.16 ± 0.83	16.98 ± 0.53	11.20 ± 0.84	< 0.001	19.67 ± 0.53	15.42 ± 0.51	13.22 ± 0.52	< 0.001
SFA	27.0 ± 0.88	23.31 ± 0.56	16.86 ± 0.89	< 0.001	25.78 ± 0.56	22.90 ± 0.54	18.44 ± 0.55	< 0.001
Cholesterol	334.42 ± 13.44	285.21 ± 8.59	211.43 ± 13.65	< 0.001	319.97 ± 8.57	289.95 ± 8.26	220.56 ± 8.38	< 0.001
Other nutrients								
Proteins (% of energy)	14.78 ± 0.22	14.10 ± 0.21	13.89 ± 0.21	0.01	15.17 ± 0.21	14.66 ± 0.20	12.98 ± 0.21	< 0.001
Fats (% of energy)	27.93 ± 0.57	27.18 ± 0.50	26.31 ± 0.51	0.46	31.27 ± 0.45	26.85 ± 0.43	22.54 ± 0.44	< 0.001
Carbohydrates (% of energy)	60.06 ± 0.63	60.66 ± 0.61	61.95 ± 0.61	0.10	55.62 ± 0.54	60.40 ± 0.52	66.41 ± 0.53	< 0.001
Dietary fiber (g/d)	21.98 ± 0.75	21.06 ± 0.48	20.51 ± 0.76	0.51	21.68 ± 0.5	21.07 ± 0.48	20.78 ± 0.49	0.43

^1^Values are Mean ± SE. Energy intake and macronutrients were adjusted for age and gender; all other values were adjusted for age, gender and energy intake.

^2^*P*-value obtained from ANCOVA test for adjustment of energy intake.

Abbreviations: *DIL* Dietary insulin load, *DII* Dietary insulin index, *T* Tertile, *SFA* Saturated fatty acids, *MUFA* Monounsaturated fatty acids, *PUFA* Polyunsaturated fatty acids.

In case of prevalence of MU among tertiles of DIL and DII, a marginally significant difference of MU in tertiles of DIL was found (T1: 40.8%, T2: 37.4 and T3: 49.2%, *P* = 0.07). The difference in prevalence of MU across tertiles of DII was not statistically significant (T1: 41.2%, T2: 39.5% and T3: 46.7%, *P* = 0.36).

Multivariable-adjusted odds ratios (and 95% CIs) for MU across tertiles of DIL and DII among all study participants are shown in Table 3. Different levels of DIL did not show a significant relationship with MU; the same finding was discovered in a linear fashion. After adjustments for all confounders, participants in the highest tertile of DII compared to the lowest had a 115% increased odds for MU (OR = 2.15, 95% CI 1.03–4.49). When the relationship was examined linearly, we found that each tertile increase in DII was associated with a 48% increase in odds of MU (OR = 1.48, 95% CI 1.02–2.14).

**Table 3:** Multivariable-adjusted odds ratios (and 95% CIs) for MU across tertiles of DIL and DII among all study participants (n = 527)^1^.

	T_1_	T_2_	T_3_	*P*_trend_	Per one tertile increase
Tertiles of energy-adjusted DIL					
MU cases/participants (n)	169/69	67/179	88/179		
Crude	1.00	0.87 (0.56–1.33)	1.40 (0.92–2.14)	0.11	1.19 (0.96–1.47)
Model 1^2^	1.00	0.73 (0.41–1.29)	1.10 (0.45–2.68)	0.97	1.01 (0.65–1.57)
Model 2^3^	1.00	1.04 (0.43–2.47)	2.84 (0.72–11.23)	0.18	1.60 (0.81–3.15)
Model 3	1.00	1.03 (0.42–2.50)	2.47 (0.62–9.91)	0.25	1.50 (0.76–2.99)
Tertiles of energy-adjusted DII					
MU cases/participants (n)	70/170	70/177	84/180		
Crude	1.00	0.94 (0.61–1.44)	1.25 (0.82–1.91)	0.29	1.12 (0.91–1.39)
Model 1	1.00	0.97 (0.62–1.54)	1.21 (0.75–1.94)	0.43	1.10 (0.87–1.40)
Model 2	1.00	1.10 (0.53–2.28)	2.21 (1.07–4.60)	0.03	1.51 (1.05–2.17)
Model 3	1.00	1.19 (0.56–2.52)	2.15 (1.03–4.49)	0.04	1.48 (1.02–2.14)

^1^All values are odd ratios and 95% confidence intervals. *P*_trend_ was obtained by the use of tertiles of DIL and DII as an ordinal variable in the model. ^2^Model 1: adjusted for age, gender, energy intake. ^3^Model 2: more adjustments for educations, marital status, smoking, socioeconomic status, physical activity levels, meal pattern, eating rate, intra meal, salt and spices. Model 3: further adjustments for BMI.

Abbreviations: *CIs* Confidence intervals, *MU* Metabolic unhealthy, *DIL* Dietary insulin load, *DII* Dietary insulin index, *T* Tertile.

Multivariable-adjusted odds ratios (and 95% CIs) for metabolic health components across tertiles of DIL and DII among all study participants are reported in Table 4. After adjustments for all confounders, individuals in the second tertile of DIL, compared to the first category, had a 156% increase in odds of hypertriglyceridemia (OR = 2.56, 95% CI 1.01–6.45). Moreover, participants in the third tertile of DII, compared to the first category, had higher odds of hypertension, both in the crude (OR = 1.52, 95% CI 1.00–2.32) and fully-adjusted model (OR = 3.57, 95% CI 1.61–7.92).

**Table 4:** Multivariable-adjusted odds ratios (and 95% CIs) for metabolic health components across tertiles of DIL and DII among all study participants (n = 527)^1^.

	Tertiles of DIL^2^				Tertiles of DII			
	T_1_ (n = 169)	T_2_ (n = 179)	T_3_ (n = 179)	P_trend_	T_1_ (n = 170)	T_2_ (n = 177)	T_3_ (n = 180)	*P*_trend_
Hyperglycemia (FBG ≥ 100 mg/dL)								
Crude	1.00	0.97 (0.57–1.66)	1.19 (0.71–2.01)	0.50	1.00	0.89 (0.52–1.50)	0.87 (0.51–1.46)	0.59
Multivariable-adjusted^3^	1.00	1.71 (0.58–5.09)	2.67 (0.51–13.95)	0.24	1.00	0.64 (0.27–1.54)	1.23 (0.54–2.82)	0.65
Hypertriglyceridemia (TG ≥ 150 mg/dL)								
Crude	1.00	1.34 (0.86–2.08)	1.37 (0.88–2.13)	0.17	1.00	1.04 (0.67–1.63)	1.42 (0.92–2.19)	0.11
Multivariable-adjusted^3^	1.00	2.56 (1.01–6.45)	1.95 (0.48–8.02)	0.30	1.00	0.97 (0.45–2.06)	1.19 (0.57–2.48)	0.63
Low HDL-cholesterolemia (< 40/50 mg/dL)								
Crude	1.00	1.19 (0.60–2.34)	1.32 (0.68–2.57)	0.42	1.00	1.58 (0.81–3.08)	1.20 (0.60–2.41)	0.64
Multivariable-adjusted^3^	1.00	2.35 (0.61–9.10)	3.62 (0.47–28.15)	0.21	1.00	2.90 (0.95–8.87)	2.14 (0.66–6.98)	0.24
Hypertension (BP ≥ 130/85 mmHg)								
Crude	1.00	0.88 (0.58–1.34)	1.21 (0.79–1.84)	0.37	1.00	1.18 (0.77–1.80)	1.52 (1.00–2.32)	0.05
Multivariable-adjusted^3^	1.00	1.70 (0.68–4.23)	2.06 (0.49–8.65)	0.30	1.00	2.08 (0.94–4.60)	3.57 (1.61–7.92)	0.01
Insulin resistance (HOMA-IR ≥ 90th)								
Crude	1.00	0.83 (0.42–1.65)	0.78 (0.38–1.56)	0.47	1.00	0.64 (0.31–1.32)	0.83 (0.43–1.64)	0.59
Multivariable-adjusted^3^	1.00	0.60 (0.17–2.17)	0.68 (0.09–5.25)	0.65	1.00	0.84 (0.28–2.58)	1.52 (0.50–4.61)	0.43
High hs-CRP (hs-CRP ≥ 90th)								
Crude		0.46 (0.22–0.95)	0.67 (0.34–1.30)	0.21	1.00	0.47 (0.22–0.97)	0.67 (0.34–1.30)	0.21
Multivariable-adjusted^3^		0.23 (0.06–0.92)	0.30 (0.04–2.25)	0.17	1.00	0.32 (0.10–1.00)	0.57 (0.22–1.50)	0.30

^1^All values are odds ratios and 95% confidence intervals.

^2^DIL and DII were adjusted for energy intake based on residual method.

^3^Adjusted for age, sex, energy intake, Education, Marital status, Smoking, socioeconomic status, physical activity levels, Meal pattern, Eating rate, Intra meal, Salt and spices, BMI.

Abbreviations: *CIs* Confidence intervals, *DIL* Dietary insulin load, *DII* Dietary insulin index, *T* Tertile, *BP* Blood pressure, *FBG* Fasting blood glucose, *TG* Triglyceride, *hs-CRP* high sensitivity C-reactive protein, *HOMA-IR* Homeostasis model insulin resistance, *HDL* High-density lipoprotein.

Multivariable-adjusted regression coefficients (B) and 95% CI for serum BDNF and adropin values per one tertile increase of DIL and DII are shown in Table 5. One tertile increase in DIL or DII was not associated with BDNF or adropin values in crude or multivariable-adjusted models.

**Table 5:** Multivariable-adjusted regression coefficients (B) and 95% CI for BDNF and adropin values per one tertile increase of DIL and DII (n = 527)^1^.

	Per one tertile increase in DIL	*P*	Per one tertile increase in DII	*P*
BDNF (ng/mL)				
Crude	− 0.04 (− 0.21,0.14)	0.66	− 0.02 (− 0.2,0.15)	0.79
Model 1^2^	− 0.03 (− 0.21,0.15)	0.75	− 0.01(− 0.19,0.18)	0.95
Model 2^3^	− 0.04 (− 0.22,0.14)	0.69	− 0.01(− 0.19,0.18)	0.97
Adropin (pg/mL)				
Crude	− 2.53 (− 6.91,1.84)	0.26	1.53 (− 2.79,5.86)	0.49
Model 1^4^	1.88 (− 6.78,10.54)	0.67	2.40 (− 2.08,6.89)	0.29
Model 2^5^	1.75 (− 5.95,9.45)	0.66	2.19 (− 1.81,6.20)	0.28

^1^All values are regression coefficient and 95% confidence intervals. P_trend_ was obtained by liner regression.

^2^Adjusted for age, sex.

^3^More adjustments for history of diabetes, hypertension, depression, physical activity level.

^4^Adjusted for age, sex, energy intake.

^5^More adjustments for physical activity level, smoking status, BMI.

Abbreviations: *CIs* Confidence intervals, *DIL* Dietary insulin load, *DII* Dietary insulin index, *BDNF* brain-derived neurotrophic factor.

## Discussion

This epidemiologic cross-sectional study demonstrated that adherence to a diet with high DII was linked to increased odds of MU in Iranian adults, but there was no significant association between DIL and metabolic health status in this population. Higher DII was additionally associated with high blood pressure. Moreover, moderate DIL was significantly associated with hypertriglyceridemia. Nevertheless, there was no significant relation between DIL and DII with serum BDNF or adropin values.

Due to the importance of the metabolic health status among both normal-weight people and individuals with overweight or obesity, community members could be clinically recommended to reduce their consumption of foods with high DII, in order to enhance their diet quality and consequently improve their health status and decrease the burden of metabolic diseases. In addition, people should be more conscious about their food choices especially the items that highly influence the insulin responses, such as refined grains, potato, sweets and desserts, and sugar.

Similar to our investigation, some previous studies have examined the association between DIL and DII with metabolic abnormalities. Mirmiran et al. in a 3-year follow-up prospective study on 927 Iranian adults have found a marginally significant association between DII and insulin resistance; greater DIL was also associated with higher risk of insulin resistance, in contrast to our null findings in case of DIL or DII and insulin resistance^15^. In addition, a cross-sectional study on data of Nurses’ Health Study (NHS) and Health Professionals Follow up Study (HPFS) has found significant positive associations between DII and DIL with low HDL-c levels in subjects with obesity and hypertriglyceridemia^27^. Moreover, Anjom-Shoae et al. have investigated the relation of DII and DIL with obesity among 8691 Iranian adults; they have found that the top quintile of DII, as compared to the bottom one, has a positive relation with general obesity in women, but not in men. However, DIL was not related to general obesity. Also, they found no significant connection between DII and DIL with central obesity^19^. These contradictory findings might be due to participants’ age range, different study design, various tools applied for dietary assessment, variation in cooking methods and meal preparation in different societies, different outcomes and variables identified as confounders, as well as various techniques for obtaining DII and DIL values.

In the current study, we found that greater tertiles of DII had a positive relation with increased odds of MU, but similar finding was not obtained for DIL. It is possible that the significant effect of DII on metabolic health status might be duo to not including energy intake and when we took energy intake into account for calculating DIL, the significant association disappeared. This could also be probable that DII exerted its effect through increasing energy intake.

The observed positive association between DII and hypertension and increased odd for hypertriglyceridemia in moderate values of DIL in the current investigation might be associated to insulinogenic effects of a diet with high DII and DIL which could result in increasing postprandial insulin and insulin resistance (IR). IR could have a key role in progression of metabolic abnormalities such as hypertension and hypertriglyceridemia^32^.

Some previous studies have investigated the relationship between dietary intakes and metabolic disorders with BDNF and adropin. Stevens et al. have observed that there was a strong relation between macronutrient intake and plasma adropin levels in lean women (with a BMI of 22–26 kg/m^2^ and age of 30–45 years). They found an inverse association between adropin values and carbohydrate intake, especially with simple sugar or unrefined carbohydrates^33^. An interventional study has reported that adherence to a Ketogenic diet could be associated with lower plasma BDNF^34^. In contrast, a randomized cross-over trial on 12 participants with MetS, with two 4-week phases and one 4-week washout period, has illustrated that a carbohydrate-restricted Paleolithic-based diet (CRPD) with exercise could increase serum BDNF levels^35^. Although several interventional investigations about the effect of diet on BDNF and adropin have been done, there are not enough population-based studies about the association between long-term dietary intakes of people with BDNF and adropin so far. Although we found that serum BDNF levels have slightly decreased by one tertile increasing in DIL and DII, this relation was not statistically significant. Also DII or DIL was not substantially linked to serum adropin values. Krabbe et al. have found that increased amounts of blood glucose and insulin resistance could prevent BDNF secretion^36^. Another study has illustrated that elevated concentration of pro-inflammatory cytokines could reduce the expression of BDNF m-RNA in hippocampus^37^. However, due to the lack of enough previous epidemiologic studies about the association of BDNF and adropin with dietary intakes in different populations, the exact reasons for our null findings between DIL and DII with BDNF and adropin were unclear.

**Figure 1:**
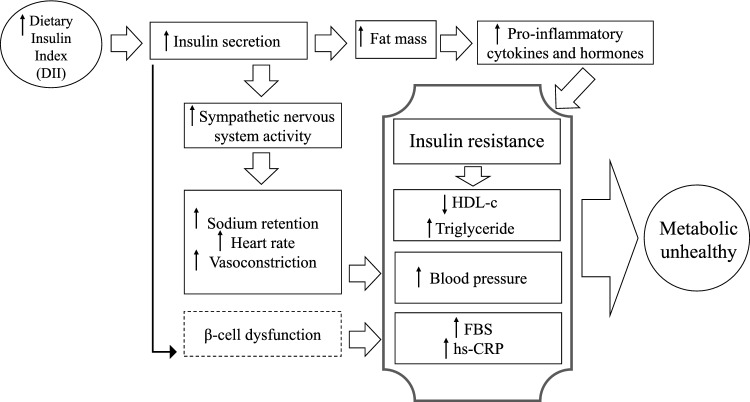
Possible mechanisms underlying the association between dietary insulin index and metabolic unhealthy.

Some basic mechanisms for explaining the association between DII and MU were suggested. As shown in Fig. 1, more intake of high potential insulinogenic foods and then greater insulin secretion in consequence for a long time could lead to accumulation of fat mass^38^ that could rise pro-inflammatory cytokines and hormones and result in IR^39^. IR could decrease activity of lipoprotein lipase (LPL) and would lead to a reduction in serum HDL-c and an increment in triglyceride concentration^40,41^. Moreover, high insulin secretion could additionally increase the activity of sympathetic nervous system which would lead to raised retention of sodium, heart rate and vasoconstriction and consequently hypertension^42^. Also, adherence to a diet that would increase postprandial insulin could be significantly related to higher FBS and hs-CRP levels, perhaps due to β-cell dysfunction^43^.

The current investigation has several advantages and limitations. We studied the relationship between DIL and DII with metabolic health status and BDNF and adropin for the first time among adults in a Middle-Eastern society. Additionally, a multistage cluster random sampling method was applied to select the study sample. It has also been tried to consider the confounding variables as complete as possible. After all, there were some limitations in our study that should be noticed. To assess dietary intake, a validated self-administered FFQ was implied; so, recall bias, misclassifications and other possible reporting biases were unavoidable and could have been an impact on the results. The residual effects of unknown or unmeasured confounders might also have an impact on the results, even after adjustments for a variety of potential confounding variables. In addition, although we tried to select a sample from all socio-economic groups or all strata of the society, due to financial limitations (in a developing country such as Iran), our sample was selected from school members. Therefore, it might not be completely representative of general Iranian adult population. The last but not the least, due to cross-sectional nature of the investigation, causality cannot be deduced. Additional researches are required to confirm the causal link.

## Conclusion

The present population-based investigation illustrated that greater DII was significantly associated with higher chance of MU and hypertension in Iranian adults; but no association was found between DIL and metabolic health. DIL and DII were not significantly linked with serum BDNF or adropin. To confirm these findings, additional researches, particularly with a prospective design, are required.

## Data Availability

The supporting data of the findings of the current study are available from the corresponding author upon request.

## Abbreviations

BDNF: Brain-derived neurotrophic factor
DIL: Dietary insulin load
DII: Dietary insulin index
MHO: Metabolically healthy obese
MUO: Metabolically unhealthy obese
MUNW: Metabolically unhealthy normal weight
MHNW: Metabolically healthy normal weight
MHOW: Metabolically healthy overweight or obese
MUOW: Metabolically unhealthy overweight or obese
HDL-c: High-density lipoprotein cholesterol
CRP: C-reactive protein
MH: Metabolically healthy
MU: Metabolically unhealthy
MetS: Metabolic syndrome
CVD: Cardiovascular disease
T2DM: Type 2 diabetes mellitus
BP: Blood pressure
FFQ: Food frequency questionnaire
24 h-DR: 24-H dietary recall,
FII: Food insulin index
BMI: Body mass index,
WC: Waist circumstance
TG: Triglyceride
FBG: Fasting blood glucose
HOMA-IR: Homeostasis model insulin resistance
IPAQ: International physical activity questionnaire
HEPA: Health-enhancing physical activity
SES: Socio-economical status
ANOVA: Analysis of variance
ANCOVA: Analysis of covariance
OR: Odd ratios
D: Decile
MUFA: Mono-unsaturated fatty acids
PUFA: Poly-unsaturated fatty acids
SFA: Saturated fatty acids
95% CI: 95% confidence interval
CRPD: Carbohydrate-restricted Paleolithic-based diet
